# A more realistic quantum mechanical model of conscious perception during binocular rivalry

**DOI:** 10.3389/fncom.2014.00015

**Published:** 2014-02-20

**Authors:** Mohammad Reza Paraan, Fatemeh Bakouie, Shahriar Gharibzadeh

**Affiliations:** ^1^Energy Engineering and Physics Department, Amirkabir University of TechnologyTehran, Iran; ^2^Neural and Cognitive Sciences Lab, Biomedical Engineering Department, Amirkabir University of TechnologyTehran, Iran

**Keywords:** consciousness, quantum state, mixed state, probability distribution, dominance duration

Since the first systematic description of binocular rivalry by Wheatstone, this fascinating phenomenon has provided several new insights into the mechanisms of visual awareness (Leopold and Logothetis, 1999). Binocular rivalry (BR) is the subjective experience of randomly alternating perceptions pertaining to the two eyes when they are presented with conflicting stimuli. Because of its nature, BR enables consciousness researchers to separately investigate the mechanisms of perception and conscious experience (Gazzaniga et al., 2009). Among various descriptions of this phenomenon, quantum mechanical descriptions stand out as the most radical.

In a recent innovative work by Manousakis, the formalism of quantum mechanics is utilized to describe the conscious experience during BR. Although the author has successfully derived the observed probability distribution of dominance durations (PDDD), his approach undermines some essential features of conscious perception during BR. Generally, two kinds of perception dominate during BR: (1) full dominance of one eye's stimulus, (2) composite or mixed dominance of the two monocular stimuli (Yang et al., 1992). Our argument revolves around the latter kind of perception which is also referred to as transition phase or transition state.

Classically, simplifications imposed experimental conditions in which only full dominance was perceived by subjects and mixed state's (MS) duration was minimized. However, many experiments reveal the diversity in rivalry's temporal dynamics and specifically the important role of MS (Hollins, 1980; Blake et al., 1992; Bossink et al., 1993; Wilson et al., 2001). Regarding the neural correlates of MS, it has been shown that the frontoparietal areas of brain trigger rivalry transitions (Lumer et al., 1998; Knapen et al., 2011). It must be emphasized that various studies on the neural concomitants of BR suggest that no single neural site or neural mechanism is at work during BR, rather multiple stages and brain areas are involved (Blake and Logothetis, 2002).

Many attempts have been made to model the dynamical behavior of BR, most of which try to reproduce the temporal dynamics of BR by reconstructing specific neural mechanisms (Kalarickal and Marshall, 2000; Laing and Chow, 2002; Stollenwerk and Bode, 2003; Freeman, 2005). A major number of these models ignore MS in order to avoid crippling complications, yet Brascamp and colleagues show that none of the previous models is capable of reproducing the full range of observed dynamics which include MS (Brascamp et al., 2006b) and hence try to develop a new model (Brascamp et al., 2006a; Noest and van Ee, 2006). Another group of models of which Manousakis' model is an example capture certain aspects of rivalry's dynamics without resorting to the underlying neural circuits (Mamassian and Goutcher, 2005). However, in order to obtain the PDDD, Manousakis employs some temporal parameters characterizing neuronal firings. This is an interesting achievement because it ties the dynamics of conscious perception to specific firing patterns.

Like the classical models, Manousakis' model only treats the two dominance states which are represented by two quantum states, while MS is ignored. The author compares his theoretical PDDD with the observed PDDD of classical experiments (Levelt, 1968; Lehky, 1995) which did not record the mixed states' duration separately. We believe that the quantum states are only symbols which are manipulated according to the quantum formalism, and bear no resemblance to the perception they represent. Therefore, in Manousakis' approach, only the number of states and their associated probabilities determine the favored PDDD. Therefore, unlike classical models, the scope of the quantum mechanical model can be readily extended by introducing a third quantum state which represents MS. In order to test the new model, its PDDD should be calculated and compared against that of experimental data which are separate recordings of dominance durations of the three states. It must be emphasized that the probability distribution is not a complete description of the dynamics of BR, and it is necessary to extract other relative quantities from the model in future works.

It is worthwhile discussing another work by Conte and colleagues who showed that mental states follow quantum mechanics during the conscious bi-stable perception of ambiguous figures (Conte et al., 2009). Their model shares a lot of features with that of Manousakis, with the exception that they take into account the periods when their subjects report indeterminate perception. Indeterminate perception resembles MS in that they are both mental states and are mediated by specific neural correlates. But Conte et al. represent indeterminacy state by the wave-function of the two-state system rather than an additional third quantum state. Technically, a wave-function is a superposition of all the real possible states of a quantum system. We believe that this is an inappropriate take on the problem which leads to inconsistencies within the model. The developers of these two quantum mechanical models believe that the actualization of each quantum state is equal to the activation of neural correlates of consciousness (NCC) of the corresponding perception; a state is actualized when a quantum system is measured (observed) and subsequently its wave-function “collapses” to that constituent state. Therefore, we believe that wave-function is not a legitimate representation, because it does not describe a real state of a system and is doomed to collapse, and on the other hand, specific NCC of MS or that of indeterminate perception demands a distinct associated quantum state.

Manousakis' neglect of MS might be justified by the presumption that this state only functions as a bridge between the two dominance states. That is, MS does not compete with the other two and is not involved in rivalry. It is noteworthy that the term “transition” has led to a misunderstanding, namely that the MS occurs only when the perception is being switched from one eye to another. But as is often the case with BR experiments, subjects report the same perception as the one that was dominant before MS. Hence, there is no particular regular periodic alternation between dominance and suppression (Mueller and Blake, 1989; Brascamp et al., 2006b). We believe these indicate that MS is not a mere bridge connecting the two dominant states, but a state which dominates consciousness randomly and therefore, enters statistical calculations of quantum mechanics.
